# Prevalence and risk factors for seclusion and restraint in old-age psychiatry inpatient units

**DOI:** 10.1186/s12888-021-03095-4

**Published:** 2021-02-08

**Authors:** Marie Chieze, Stefan Kaiser, Delphine Courvoisier, Samia Hurst, Othman Sentissi, Jérôme Fredouille, Alexandre Wullschleger

**Affiliations:** 1 Adult Psychiatry Division, Department of Psychiatry, University Hospital of Geneva, Chemin du Petit-Bel-Air 2, 1226 Thônex, Switzerland; 2 Quality of Care Service, University Hospital of Geneva, Geneva, Switzerland; 3 Institute for Ethics, History and the Humanities, Faculty of Medicine, University of Geneva, Geneva, Switzerland; 4 Geriatric Psychiatry Division, Department of Psychiatry, University Hospital of Geneva, Geneva, Switzerland

**Keywords:** Coercion, Restraint, Seclusion, Geriatric psychiatry, Risk factors

## Abstract

**Background and objectives:**

Coercion in psychiatry is legally tolerated as a last resort. The reduction of the use of coercion is a shared goal of hospital administrators, medical and nursing staff and representatives of patients and families but requires the identification of risk factors for coercion. These risk factors in geriatric psychiatric inpatient settings are not well known, especially regarding seclusion. Through examining the prevalence of coercion and patients’ characteristics, this study aims to identify risk factors for coercion in elderly people.

**Methods:**

The use of coercion in the geriatric psychiatry division of Geneva University Hospital in 2017 was retrospectively analyzed. The incidence rate ratios were estimated with multivariable Poisson regressions to assess risk factors for coercion.

**Results:**

Eighty-one of 494 patients (16.4%) experienced at least one coercive measure during their stay (mainly seclusion). The risk factors for coercion were younger age, male gender, being divorced or married, cognitive disorders, high item 1 of the Health of the Nation Outcome Scales (HoNOS) score (overactive, aggressive, disruptive or agitated behavior) at admission, previous psychiatric hospitalizations and involuntary referrals from the emergency department. Other disorders and global HoNOS scores were not associated with the use of coercion.

**Conclusion:**

Higher risks of coercion were outlined in men with cognitive disorders, agitated behaviors, and previous psychiatric hospitalizations. They differed from those observed in younger adults in terms of age, civil status, disorders, global HoNOS scores and referrals. Therefore, geriatric psychiatric populations should be specifically investigated for the development of interventions aiming coercion reduction.

## Introduction

Coercion – defined as any intervention limiting a patient’s choice, autonomy or liberty of movement [1] – infringes upon fundamental human rights and therefore highlights legal and ethical issues [2, 3]. The use of coercion, which is legally tolerated as a last resort [2], is common in psychiatry, ordinarily to manage aggression and violence [4, 5]. The use of coercion is peculiarly contentious, as mental disorders can temporarily impair judgment capacity and hence make patients particularly vulnerable [6]. Because of the potential deleterious consequences of coercion, the worldwide trend is to prevent or at least diminish the use of coercion in psychiatry [7–10]. The main aim is to reduce the prevalence and duration of seclusion and restraint [11].

Among the general psychiatric population experiencing coercive measures, the psychogeriatric population has been rarely studied to date. Moreover, the interpretation of the results of studies into clinical practice in this population are particularly difficult due to the methodological heterogeneity of the available studies, for example, regarding ages of inclusion [12, 13] or settings [14–16]. Although restraint is commonly the main analyzed coercive measure in elderly people [12, 17], the wide heterogeneity of practices used in this population have resulted in wide definitions of restraint, including, for example, the use of bedrails [18, 19]. Restraint prevalence in elderly people varies between 7.4 and 85% [20, 21]. The discrepancy depends mainly on the definition used for restraint and the study setting [22]. As a comparison, in general adult hospital care, the prevalence of restraint usually trends from 6 to 17% [23, 24]. To our knowledge, no study has specifically examined the use of seclusion in elderly people. A comparison of results could thus be problematic, as in Switzerland, as opposed to other countries, restraint is rarely used compared to seclusion [25]. These methodological issues render the interpretation of available data about the prevalence of coercive measures difficult, particularly considering the often contrasting results [21].

Regarding the risk factors for coercion in elderly people, it is important to underline that most studies do not specifically address the question of coercion in specific populations, such as elderly people; they only examine younger adult psychiatric populations [26, 27] or make no distinction between adult and geriatric populations [28, 29]. Some studies exclude patients with cognitive impairments [30, 31], which are preponderant in elderly people [18, 32]. In some studies, the relevant risk factors for coercion are organic mental disorders [33–35], older age [36, 37] and physical complications [38], three features associated with older populations. These results suggest a possible substantial difference in terms of the risk factors for coercion between adult and geriatric populations that needs to be investigated [12]. The inconsistency of the literature regarding this theme could thus come from the nondistinction between two noncomparable populations [25]. In the few studies available specifically concerning the risk of coercion in elderly people, the main risk factors for restraint (with or without seclusion) in elderly people were older age, cognitive impairment, disruptive behaviors (confusion, agitation, regression, screaming), wandering, high dependency in activities of daily living, reduced mobility, and history of falls [15, 16, 32]. Gender was usually not associated with the risk of coercion [12, 17]. The main reasons for using restraint were the prevention of falls, injuries or wandering; protection of medical devices; and management of disruptive behaviors [20, 39]. Aggression and violence were less common [14, 40], which contrasts with the adult psychiatric population in which aggressive behavior represents the main reason for using coercion [4, 5]. These discrepancies are arguments in favor of the significant differences between the two populations. As the clinical management of patients exposed to coercion may differ depending on the risk factors, those differences need to be assessed.

This study aims to determine the risk factors for coercion in the psychogeriatric population and to analyze its prevalence. Based on a previous study on patients hospitalized in the adult psychiatry division of Geneva University Hospital during 2017, this work also aims to compare the risk factors for coercion between geriatric and adult psychiatric populations [41]. This study should, therefore, help determine whether specific interventions should be implemented in psychogeriatric settings to reduce the use of coercion.

## Material and methods

The present study uses the same retrospective design as described in a previous work [41].

### Participant selection

Data on patients’ sociodemographic and clinical features, hospital stays, and coercion prescriptions were retrospectively collected from electronic patient files and anonymized.

Patients hospitalized in the geriatric psychiatry division of the Geneva University Hospital between 1 January and 31 December 2017 were included. Patients admitted before 1 January or discharged after 31 December 2017 were also included.

The four wards of this division offer inpatient care for patients older than 65 years. Three of these units provide acute care; one of them is specifically dedicated to patients with cognitive disorders. The doors of these three wards are continuously closed. The fourth unit provides postacute care and conforms to an open-door policy, with the main door being opened from 8 am to 8 pm and without special surveillance of the ward exit. Opening on request is available from 8 pm to 8 am. Concerning the use of coercion, the division’s guidelines prioritize the use of seclusion and forced medication rather than a four- or five-point mechanical restraint, which is only applied in extraordinary circumstances. Other forms of restraint are occasionally used in our geriatric psychiatry division, such as seatbelts, holding seat tables, and abdominal bed holding belts. Coercion also includes bedrails and patient antiwandering devices (alarm mats, wristband transmitters).

### Data collection

Since 1 January 2017, coercive measures – clustered as seclusion, restraint (five-point belts, immobilization, seatbelts, ankles and wrists fasteners, chair tables, waist-belts, bedrails) or other forms (forced medication, manual restraint, anti-wandering devices) – have been directly prescribed in patients’ electronic health records. We automatically extracted the number of times coercive measures were prescribed from these electronic files. This extraction, due to the way the prescriptions were made, did not permit to distinguish between emergency forced medication and forced treatment under Article 434 of the Swiss Civil Code (dispensed outside of an acute emergency in case of a severe threat to the patient’s health or others’ life or integrity without treatment) [42]. We adopted the occurrence of at least one coercive measure as the main (dependent) outcome. When seclusion and restraint were both used during one hospital stay, we decided to allocate them to the restraint cluster, as it is regularly reported as more constraining and traumatic than seclusion [43–45]. The literature also suggests studying the combination of different coercive measures [46], but the small number of restraint measures did not allow for cluster analyses.

Some patients were hospitalized several times during the year and/or were prescribed several coercive measures during one hospital stay, meaning the data were dependent. Thus, we distinguished patient-related from stay-related variables.

Gender, age, civil status (single, married, separated/divorced, widowed), nationality (Swiss/foreign), number of previous stays during the year (1, 2, 3 and more), and the presence (yes/no) and number of previous psychiatric hospitalizations were considered patient-related variables. Stay-related variables included the source of the hospitalization decision (outpatient center or private physician practicing outside of the hospital, hospital physician, emergency department, other), main diagnosis (organic/neurologic (F0/G2-G3) [47], psychotic (F2), bipolar (F30–31), depressive (F32–33), personality (F6), anxious and behavioral disorders (F4-F5), substance use (F1), other diagnoses (developmental (F7-F8) and other)), number of days spent in the hospital in 2017 and admission status (voluntary/involuntary). The Health of the Nation Outcome Scales (HoNOS) scores at admission and discharge were examined to assess the burden of symptoms [48]. The first item on this scale rates symptoms of overactive, aggressive, disruptive or agitated behaviors. This item was, therefore, chosen to analyze the influence of violence on the use of coercion.

### Data analysis

For the descriptive analyses, a non-normal distribution was presumed for quantitative variables, and a Kruskal-Wallis rank-sum test was performed to compare groups. Regarding the qualitative variables, expected frequencies higher or lower than five determined the use of Pearson’s chi-squared test or Fisher’s exact test, respectively.

To identify the risk factors for coercion, we used multivariable Poisson regressions. When there were missing data, multiple imputations with chained equations were performed (50 imputations sample). The global incidence rate (IR) represented the number of hospitalization days with at least one coercive measure out of 365 hospitalization days. The significant variables from the descriptive analyses were used to obtain incidence rate ratios (IRRs) (or the ratio of the rate of coercion prescriptions per timeframe). IRRs significantly higher (or lower) than 1 in the exposed cluster indicate an increased (or reduced) risk of coercion. Nonsignificant or potentially redundant variables were not retained for the multivariable analyses (the HoNOS scores at discharge, number of previous stays during the year, existence of previous psychiatric hospitalizations, nationality).

R software for statistics, version 3.6.1, was used for statistical analyses. The significance threshold was *P* < 0.05.

### Human participant protection

The study was approved by the Swiss Ethics Committee on Research Involving Humans of Geneva (No. 2018–00988).

## Results

### Descriptive analyses (Table 1)

In 2017, 16.4% (*n* = 81) of the patients hospitalized in Geriatric Psychiatry experienced at least one coercive measure. At least one coercive measure was prescribed in 16.8% (*n* = 102) of the hospital stays. At the hospital stay level, seclusion was the most prescribed coercive measure (77.4%), followed by restraint (16.7%). Forced medication or other coercive measures accounted for 5.9% of the prescribed measures. Restraint prescriptions included bedrails (*n* = 6; 35.3%), chair-tiding (*n* = 6; 35.3%), bed-tiding (*n* = 4; 23.5%) and immobilization (*n* = 1; 5.9%).

**Table 1:** Descriptive analyses

	No Coercion	Coercion	Test	*p*-value
**Patient-related Variables**				
*N* = 494 (%)	413 (83.6)	81 (16.4)		
Gender = male (%)	158 (38.3)	49 (60.5)	12.86^a^	**< 0.001**
Age (year) (median [IQR])	77.00 [70.00, 84.00]	79.00 [73.00, 84.00]	3.44^b^	0.064
Civil status (%)			**12.53**^a^	**0.006**
Single	77 (18.6)	8 (9.9)		
Married living as a couple	141 (34.1)	43 (53.1)		
Separated/divorced	103 (24.9)	12 (14.8)		
Widowed	92 (22.3)	18 (22.2)		
Nationality = Swiss (%)	313 (75.8)	53 (65.4)	3.26^a^	0.071
No. of hospital stays in 2017 (%)			Fisher^c^	0.30
1	345 (83.5)	63 (77.8)		
2	51 (12.3)	15 (18.5)		
3+	17 (4.1)	3 (3.7)		
Total no. of psychiatric hospitalizations (median [IQR])	0.00 [0.00, 1.00]	0.00 [0.00, 1.00]	0.0085^b^	0.93
Previous psychiatric hospitalization = yes (%)	165 (40.0)	31 (38.3)	0.025^a^	0.87
Total hospitalization duration in 2017 (days) (median [IQR])	35.82 [17.70, 66.47]	58.10 [25.91, 111.01]	**14.27**^b^	**< 0.001**
**Hospital stay-related Variables**				
*N* = 606 (%)	504 (83.2)	102 (16.8) (79 seclusion (77.4) 17 restraint (16.7) 6 else (5.9))		
Hospitalization decision (%)			Fisher^c^	0.20
Outpatient center or private physician	203 (40.3)	31 (30.4)		
Hospital physician	113 (22.4)	23 (22.5)		
Emergencies	183 (36.3)	47 (46.1)		
Other	5 (1.0)	1 (1.0)		
Main diagnosis (%)			**Fisher**^c^	**< 0.001**
Organic/neurologic disorders	139 (31.2)	30 (41.7)		
Substance use	10 (2.2)	2 (2.8)		
Psychotic disorders	55 (12.3)	8 (11.1)		
Bipolar disorders	43 (9.6)	17 (23.6)		
Depressive disorders	127 (28.5)	6 (8.3)		
Anxious and behavioral disorders	51 (11.4)	4 (5.6)		
Personality disorders	16 (3.6)	2 (2.8)		
Other	5 (1.1)	3 (4.2)		
Involuntary admission = yes (%)	260 (51.6)	76 (74.5)	**17.13**^a^	**< 0.001**
Admission HoNOS (median [IQR])	17.00 [11.00, 22.00]	21.50 [15.25, 27.00]	**24.45**^b^	**< 0.001**
Discharge HoNOS (median [IQR])	9.00 [5.00, 14.00]	12.00 [6.00, 19.75]	**10.01**^b^	**0.002**
Admission HoNOS item 1 (median [IQR]) (overactive, aggressive, disruptive or agitated behavior)	1.00 [0.00, 3.00]	3.00 [2.00, 4.00]	**53.09**^b^	**< 0.001**
Discharge HoNOS item 1(median [IQR])	0.00 [0.00, 0.00]	1.00 [0.00, 2.00]	**35.10**^b^	**< 0.001**
Stay duration (days) (median [IQR])	29.92 [15.74, 54.59]	52.22 [24.63, 94.17]	**23.37**^b^	**< 0.001**

Abbreviations: *IQR* Interquartile Range, *No.* Number, *HoNOS* Health of the Nation Outcome Scales

^a^Pearson’s Chi-squared test; ^b^Kruskal-Wallis rank-sum test; ^c^Fisher’s exact test

Group comparisons showed that men (*n* = 49 (60.5%) vs. *n* = 158 (38.3%)) as well as married patients (43 (53.1%) vs. 141 (34.1%)) were overrepresented among patients who experienced coercion. Patients experiencing coercion also spent more time in the hospital in 2017 (58.10 vs. 35.82 days). Considering clinical factors, organic (*n* = 30 (41.7%) vs. *n* = 139 (31.2%)) and bipolar (*n* = 17 (23.6%) vs. *n* = 43 (9.6%)) disorders were overrepresented among stays with at least one coercive measure. Involuntary admission was more common in stays with coercion (*n* = 76 (74.5%) vs. *n* = 260 (51.6%)). The mean duration of stay was also longer when at least one coercive measure occurred (52.22 vs. 29.92 days). The mean global and item 1 HoNOS scores at admission and discharge were higher in cases of coercion (global score at admission: 21.5 vs. 17.0; at discharge: 12.0 vs. 9.0; item 1 score at admission: 3.0 vs. 1.0; at discharge: 1.0 vs. 0.0).

### Multivariable analyses (Table 2)

The global incidence rate (IR) was 12.5 per hospital stay year, meaning that from a total of 365 days of hospitalization, coercion was prescribed on 12.5 days on average (95% CI [11.8, 13.3]).

**Table 2:** IRR per geriatric psychiatric hospital stay per year with multiple imputations

	IRR	95% CI	*p*-value
Gender = male	3.13	[2.77, 3.77]	**< 0.001**
Age (year)	0.96	[0.96, 0.96]	**< 0.001**
Civil status			
Single	1		
Married living as a couple	1.62	[1.25, 1.90]	**< 0.001**
Separated/divorced	2.04	[1.63, 2.51]	**< 0.001**
Widowed	1.11	[0.81, 1.49]	0.50
Total no. of psychiatric hospitalizations	1.06	[1.06, 1.07]	**< 0.001**
Hospitalization decision			
Outpatient center or private physician	1		
Hospital physician	0.99	[0.77, 1.26]	0.96
Emergencies	2.82	[2.43, 3.44]	**< 0.001**
Other	0.54	[0.08, 1.56]	0.40
Main Diagnosis			
Depressive disorders	1		
Organic/neurologic disorders	1.30	[1.27, 1.34]	**0.026**
Substance use	0.46	[0.18, 0.53]	**0.0037**
Psychotic disorders	0.60	[0.33, 0.68]	**0.0067**
Bipolar disorders	1.01	[0.96, 1.75]	0.93
Anxious and behavioral disorders	0.49	[0.15, 0.53]	**0.0099**
Personality disorders	0.66	[0.64, 0.68]	**0.011**
Other	1.19	[0.63, 2.72]	0.63
Involuntary admission = yes	2.88	[2.25, 3.22]	**< 0.001**
Admission HoNOS	0.99	[0.99, 1.01]	0.20
Admission HoNOS item 1 (overactive, aggressive, disruptive or agitated behavior)	1.39	[1.23, 1.41]	**< 0.001**

Abbreviations: *IRR* Incidence Rate Ratio, *CI* Confidence Intervals, *No.* Number, *HoNOS* Health of the Nation Outcome Scales

#### Demographic risk factors

After adjusting for other variables, men were shown to be at higher risk of being subject to coercion than women (IRR 3.13 [2.77, 3.77]). Age was associated with a reduced risk of coercion (IRR 0.96 [0.96, 0.96]). The risk of coercion was higher in separated/divorced and married patients living as a couple than in single patients (IRRs 2.04 [1.63, 2.51] and 1.62 [1.25, 1.90], respectively). The risk of coercion increased with the number of previous psychiatric hospitalizations (IRR 1.06 [1.06, 1.07]).

#### Clinical risk factors

Compared to referrals from an outpatient physician, being hospitalized from the emergency department was associated with a higher risk of coercion (IRR 2.82 [2.43, 3.44]). The risk of coercion was significantly higher in diagnoses of organic disorders than depressive disorders (IRR 1.30 [1.27, 1.34]). A reduced risk of coercion was observed in diagnoses of substance use as well as psychotic, anxious and behavioral and personality disorders (IRRs 0.46 [0.18, 0.53], 0.60 [0.33, 0.68], 0.49 [0.15, 0.53], 0.66 [0.64, 0.68], respectively). Bipolar disorders were not significantly associated with a risk of coercion compared to depressive disorders. The risk of coercion was higher in case of involuntary admission (IRR 2.88 [2.25, 3.22]) and was correlated with higher item 1 HoNOS scores at admission (IRR 1.39 [1.23, 1.41]). The Global HoNOS scores at admission were not associated with the risk of coercion.

The main findings are summarized in Table 3.

**Table 3:** Key messages

**Descriptive analyses**	
- 16.4% of the geriatric psychiatric patients with at least one coercive measure Compared to non-coerced patients:	
- Increased hospitalization duration	
- Significantly more likely to be male	
- Significantly different civil status (mainly married patients living as a couple (53.1%))	
- 16.8% of hospital stays with at least one coercive measure, mainly seclusion (77.4%)	
- Organic (41.7%) and bipolar (23.6%) disorders as the most frequent diagnoses	
- Hospitalization decision mostly originated from the emergency department (46.1%) in case of hospital stays with coercive measures	
- Higher global and item 1 admission HoNOS scores	
**Multivariable analyses**	
Increased risk of coercion in:	
- Men	
- Separated/divorced or married patients living as a couple compared to single patients	
- Higher number of previous psychiatric hospitalizations	
- Higher item 1 admission HoNOS scores (overactive, aggressive, disruptive or agitated behavior)	
- Organic disorders diagnosis compared to depressive disorders	
- Hospitalization decision from the emergency department compared to an outpatient center’s or private physician’s decision	
Reduced risk of coercion in:	
- Older age	
- Diagnoses of substance use as well as psychotic, anxious and behavioral, and personality disorders compared to a depressive disorder	
Global admission HoNOS scores were not a significant risk factor for coercion, nor was a diagnosis of bipolar disorder compared to a depressive disorder or being widowed compared to being single.	

## Discussion

In 2017, 16.4% of patients experienced at least one coercive measure during their hospitalization in geriatric psychiatry. Considering demographic factors, the risk of coercion was correlated with male gender, younger age and a history of previous psychiatric hospitalizations. Separated/divorced or married patients were at higher risk of coercion than single patients. Regarding clinical risk factors, referrals from the emergency department, involuntary admission, high item 1 HoNOS scores at admission and a diagnosis of cognitive disorder were associated with a higher risk of coercion. Diagnoses of psychotic, anxious or personality disorders were associated with a lower risk of coercion. This risk was not influenced by a diagnosis of bipolar disorder or the global HoNOS scores at admission (Table 3).

The prevalence of the patients experiencing coercion in our geriatric psychiatric division was 16.4%, which is consistent with the literature, as the known proportion is approximately 7.4–20% in acute geriatric care hospitals [21]. This result is also similar to our findings in the nongeriatric adult population at the same hospital [41].

Male gender was associated with a higher risk of coercion in our sample than female gender, a finding that differs from previous psychogeriatric studies [12, 17] but is consistent with our findings among the adult psychiatric population [41]. It is possible that men exhibit more violent behaviors and/or induce more fear in staff.

The risk of coercion decreased with age in our sample, which diverges from previous works identifying older age as a risk factor for restraint in the geriatric population [12, 15]. In adult populations and similar to our present study, younger age was correlated with an increased risk of coercion in some publications [24, 49], whereas other studies, including our previous work, found no association between age and coercion in adults [25, 41, 50]. In younger patients, coercion is mostly used to manage aggression and violence [4, 5], whereas in elderly patients, the main reasons for coercion seem to be disruptive behavior and fall prevention, often in association with cognitive disorders [16, 39]. The same intervention – coercion – seems therefore to be used for two different purposes, suggesting that there are two substantially distinct populations that need to be studied separately.

In our study, divorced or married patients were at higher risk of coercion than single patients. Reliable inferences at this stage are difficult to establish, with highly divergent results in the literature [24, 45, 51]. As a comparison, we found a lower risk of coercion in married or divorced adult patients [41]. Civil status in elderly people seems differently associated with coercion compared to that in adults. A hypothesis could be that cognitive disorders can have behavioral disturbances with a relational manifestation, such as agitation and aggression with relatives. These symptoms may lead to coercion. Another hypothesis could be that single patients more often live in protected environments or in nursing homes and therefore require less hospital care.

The risk of coercion increased with the number of previous psychiatric hospitalizations, suggesting a higher risk of coercion in cases with more severe disorders [50, 52]. This result is similar to our findings in adults [41].

Consistent with other studies, our results showed that cognitive disorders were the only diagnosis-related risk factor for coercion in geriatric psychiatric populations – using depressive disorders for comparison [12, 53]. Cognitive disorders are indeed more common in elderly people and alter their judgment capacity as well as their behavior, leading to the need for coercion. Opposite to what was found in adult populations, diagnoses of psychotic or bipolar disorders were not associated with a higher risk of coercion in this population [25, 43, 54]. Moreover, our previous results in adults showed a higher risk of coercion among patients suffering from substance use and personality disorders, whereas these risks were reduced in geriatric patients [41]. Patients suffering from substance use or a personality disorder tend to present less aggressive symptoms when their age increases [55, 56].

Referrals from the emergency department were associated with an increased risk of coercion in elderly people. Confusional states can lead to disruptive behaviors and thus to coercion [18, 32]. In such states, the somatic etiology needs to be excluded, which could explain the visit to the emergency service before hospitalization in geriatric psychiatry. A similar rationale could be applied to falls and the need for a somatic examination as well as the use of coercion as a prevention during hospitalization. Other studies in adult populations have reported comparable results [25, 57]. Our study in adults, however, showed no association with referrals from the emergency department [41].

In this study, the risk of coercion increased with the item 1 rating on the HoNOS at admission. This result was similar in adults [41]. Despite the discrepancies in disorders impacting the risk of coercion differently between the two populations, the symptoms rated by the first item of the HoNOS (overactive, aggressive, disruptive or agitated behaviors) seem to be good predictors of the risk of coercion for both populations and should thus be systematically evaluated in practice. The global HoNOS scores at admission were however not significantly associated with a risk of coercion in elderly people. Another study showed that the HoNOS score was not predictive of the use of seclusion in cases of cognitive disorders [58], whereas the global admission scores were predictors of coercion in adults [41]. Cognitive disorders, which are prevalent in elderly people could thus hinder the pertinence of the global HoNOS to predict the risk of coercion in psychogeriatric populations.

### Implications for clinical practice

Decreasing the use of coercion in elderly people requires an awareness of the associated specific risk factors. This awareness can serve in clinical practice as an indicator for patients who require special attention to avoid coercion. It should also lead to the development of interventions tailored to deal with these specific clinical factors. The present work should be considered a first step towards the implementation of such new interventions.

As mentioned before, the lack of publications focusing on seclusion – the most used coercive measure in our hospital – in this population renders comparisons between studies somewhat difficult. We can still contrast some of our results with the known literature, as parallels between restraint and seclusion can be drawn. Prevention of falls and injuries and management of disruptive behaviors are the principal reasons for using restraint in elderly people [17, 59]. As a parallel, the present study shows that the risk factors for seclusion are mainly cognitive disorders and agitated behaviors. Restraint is also known to be a risk factor for confusion, agitation, and risk of falls, reasons often evoked to justify its use [40, 60, 61]. Similarly, it can be clinically argued that secluding a patient suffering from cognitive disorders could lead to the risk of increasing confusion and agitation through loss of orientation and isolation. These two coercive methods seem, therefore, to have similar risk factors and side effects and might not be the most appropriate to treat elder patients with cognitive impairment [32, 60].

Alternatives and oriented interventions to decrease the use of coercion in the older population are thus more than needed [18, 62]. Interventions directly targeting the symptoms of disorientation and/or derealization that increase the risk of disruptive behaviors among patients suffering from cognitive impairments might be interesting and promising alternatives. For example, architectural changes in wards, such as multisensory rooms or senses-based interventions, including Snoezelen therapy or a “controlled multisensory environment,” aimed at alleviating the symptoms of disorientation and/or derealization through sensory stimuli seem promising [63, 64]. Including a patient’s relatives in clinical discussions and decisions is also an alternative for the care of patients with cognitive impairments [61, 65]. Regarding staff, some studies examining geriatric care have found that specific staff training in geriatrics and psychiatry sensitizes nurses to cognitive impairment management and thus helps reduce the use of restraint [16, 39].

### Strengths and limitations

To our knowledge, the present study is the first to analyze the risk factors of coercion in geriatric psychiatric units using mainly seclusion. The relevant demographic and clinical features that contribute to the use of coercion are emphasized here, which will help develop oriented interventions aiming at reducing coercion. This study on elderly patients follows a similar one in adults using the same methodology. Comparison between the two studies is, therefore, reliable and brings to light significant differences in the risk factors for coercion between the two populations.

The limitations of this study involve the accessibility of some data concerning staff- and environmental-related levels of risk factors for coercion. Regarding patient-related factors, some variables could be of interest, such as the level of education, spoken language and origin, but these data were not yet available. The descriptive analysis suggests that Foreign compared to Swiss nationality may be associated with an increased risk for coercion. However, this result should be interpreted was caution, because the effect was small and reached only trend-level significance. Furthermore, in order to contextualize the nationality status information on spoken language and origin would be needed, which were not available in our database. Another patient-related variable that was not available concerns size and physical stature. A recent study on nurses suggests that differences in stature between patients and nurses may have an impact on the feeling of safety and use of coercive measures [66]. Data regarding the staff- and institutional-related variables were not included in this study, first due to data availability and second because the present work mainly focuses on patients’ characteristics. Future studies are planned to specifically investigate the role of these variables, including staff/patient ratios, day and time (nights and weekends) of coercion prescriptions.

## Conclusions

The present work outlined higher risk of coercion among men with cognitive disorders, agitated behaviors, and previous psychiatric hospitalizations. It also highlighted the differences in the use of coercion compared to younger adults, especially regarding age, civil status and diagnostic and clinical factors. These results support the specificities of the geriatric psychiatric population and indicate the need for further research investigating the clinical processes leading to the use of coercion in the elderly. This study clearly states that specific clinical interventions are needed to offer alternatives in the management of critical situations in this population and to effectively reduce the use of coercion.

## Data Availability

The anonymized data and the research protocol that support the findings of this study are available on request from the corresponding author, MC. The data are not publicly available due to their containing information that could compromise the privacy of research participants.

## Abbreviations

CI: Confidence intervals
HoNOS: Health of the Nation Outcome Scales
IQR: Interquartile range
IRR: Incidence rate ratio
No.: Number
Vs.: Versus
